# The Effect of Zirconium on the Microstructure and Properties of Cast AlCoCrFeNi_2.1_ Eutectic High-Entropy Alloy

**DOI:** 10.3390/ma17235938

**Published:** 2024-12-04

**Authors:** Rongbin Li, Weichu Sun, Saiya Li, Zhijun Cheng

**Affiliations:** 1 School of Materials Science and Engineering, Shanghai Dianji University, Shanghai 201306, China; 23600005130126@st.sdju.edu.cn; 2 School of Materials and Chemistry, University of Shanghai for Science and Technology, Shanghai 200093, China; 223353181@st.usst.edu.cn (W.S.); 223353217@st.usst.edu.cn (Z.C.)

**Keywords:** Zr_x_AlCoCrFeNi_2.1_ high-entropy alloy, zirconium content, creep, mechanical properties, corrosion

## Abstract

To improve the performance of AlCoCrFeNi_2.1_ eutectic high-entropy alloys (EHEA) to meet industrial application requirements, Zr_x_AlCoCrFeNi_2.1_ high-entropy alloys (x = 0, 0.01, 0.05, 0.1) were synthesized through vacuum induction melting. Their microstructures were analyzed using X-ray diffraction (XRD), scanning electron microscopy (SEM), and energy-dispersive spectroscopy (EDS). Additionally, the hardness, low-temperature compressive properties, nanoindentation creep behavior, and corrosion resistance of these alloys were evaluated. The results showed that AlCoCrFeNi_2.1_ is a eutectic high-entropy alloy composed of FCC and B2 phases, with the FCC phase being the primary phase. The addition of Zr significantly affected the phase stability, promoting the formation of intermetallic compounds such as Ni_7_Zr_2_, which acted as a bridge between the FCC and B2 phases. Zr addition enhanced the performance of the alloy through solid-solution and dispersion strengthening. However, as the Zr content increased, Ni gradually precipitated from the B2 phase, leading to a reduction in the fraction of the B2 phase. Consequently, at x = 0.1, the microhardness and compressive strength decreased at room temperature. Furthermore, a higher Zr content reduced the sensitivity of the alloy to loading rate changes during creep. At x = 0.05, the creep exponent exceeded 3, indicating that dislocation creep mechanisms dominated. In the Zr_x_AlCoCrFeNi_2.1_ (where x = 0, 0.01, 0.05, 0.1) alloys, when the Zr content is 0.1, the alloy exhibits the lowest self-corrosion current density of 0.034197 μA/cm^2^ and the highest pitting potential of 323.06 mV, indicating that the alloy has the best corrosion resistance.

## 1. Introduction

In recent years, high entropy alloys (HEAs) [1], also referred to as multicomponent alloys (MCAs) [2], have increasingly been explored as potential materials for various applications due to their outstanding properties, such as high strength [3], excellent plasticity [4], resistance to oxidation [5], and superior corrosion resistance [6]. HEAs generally comprise at least five principal elements, creating a homogeneous solid solution within a high-entropy state through the combination of multiple elements. This complex composition and structure result in numerous dislocation defects and grain boundaries in the material, which effectively restrict dislocation movement and enhance the material’s strength and ductility [7,8]. However, balancing strength and ductility in single-phase HEAs remains a challenge [9]. To address this issue, Lu et al. [10]. from Dalian University of Technology introduced an AlCoCrFeNi_2.1_ eutectic high entropy alloy in 2014, featuring a uniform, fine lamellar eutectic structure similar to pearlite. This alloy offers a novel approach for producing large-sized high-entropy alloys. Despite having good strength and plasticity, the AlCoCrFeNi_2.1_ eutectic high-entropy alloy still does not fully meet industrial application standards, underscoring the need for further performance enhancements to expand its practical utility. Nevertheless, the performance of the AlCoCrFeNi_2.1_ eutectic high-entropy alloy still falls short of the industrial application requirements, making it crucial to further enhance its performance to broaden its practical application potential. Zirconium (Zr) is often added as a solute atom in high-entropy alloy systems. Research has shown that Zr can induce the formation of hard Laves phases, thereby improving the compressive performance of Al_2_NbTi_3_V_2_Zr_x_ alloys [11]. Additionally, adding Zr to CoCrFeNiNb entropy alloys can significantly refine the grain structure and increase the grain boundary density, which contributes to enhancing the yield strength and hardness of the alloy [12].

In aircrafts and spacecraft, fasteners are critical components that ensure the safe connection of various parts. These fasteners must withstand extreme temperature fluctuations and endure long-term mechanical loading. In such environments, room-temperature creep and corrosion resistance are significant factors that affect the long-term stability and lifespan of fasteners. Currently, research on the creep and corrosion resistance of eutectic high-entropy alloys is limited owing to the sample size and experimental environment constraints, hindering their widespread application in the field. To provide more references for the creep performance testing of eutectic high-entropy alloys, this study employed nanoindentation techniques as a substitute for traditional uniaxial tensile or compression tests to investigate their creep behavior and corrosion resistance at the nanoscale. Existing studies have shown that high-entropy alloys with added Zr exhibit improved creep resistance. For instance, the AlNbTiVZr high-entropy alloy shows enhanced hardness and reduced plasticity owing to the solid-solution strengthening effect of Zr and the formation of second-phase particles at the grain boundaries, thereby improving creep resistance [13]. Additionally, Zr can stabilize the crystal structure of TaNbHfZrTi high-entropy alloys, particularly their face-centered cubic (FCC) structure. A stable crystal structure is less likely to undergo phase transformations or grain boundary sliding at high temperatures, thus enhancing the creep resistance [14]. Despite the attention given to AlCoCrFeNi_2.1_ eutectic high-entropy alloys owing to their excellent properties, research on the influence of Zr on their performance remains limited. In this study, Zr_x_AlCoCrFeNi_2.1_ (x = 0, 0.01, 0.05, 0.1) high-entropy alloys were prepared using a vacuum induction melting method. The changes in the microstructure of the alloys after the addition of Zr were investigated through XRD, SEM, and TEM. The hardness, room temperature creep resistance, room temperature compressive properties, and corrosion resistance of the Zr_x_AlCoCrFeNi_2.1_ (x = 0, 0.01, 0.05, 0.1) high-entropy alloys were studied.

## 2. Materials and Methods

In this experiment, using high-purity aluminum, cobalt, chromium, iron, nickel, and zinc ingots (99.99 wt%) from Zhongnuo New Materials Technology Co., Ltd. (Beijing, China), the materials were proportioned according to atomic percentages and accurately weighed using an electronic balance. Under argon protection, Zr_x_AlCoCrFeNi_2.1_ (x = 0, 0.01, 0.05, and 0.1) alloys were produced using cold crucible floating melting. To ensure chemical homogeneity, each alloy was remelted at least five times and the purity of the alloying elements was maintained at no less than 99.9 wt%; among them, the cast ingot had a diameter of 44 mm and was in the shape of a button. The nominal and actual compositions of the alloys are listed in Table 1, which shows that the components are generally consistent within the error range.

This study employed a Bruker D8 X-ray diffractometer (XRD) (Bruker Corporation, Karlsruhe, Germany) equipped with a Cu target for phase analysis with a scanning range of 20° to 95°. The Jade 6 software was used to calculate the lattice parameters of the phases in the alloy. Microstructural characterization and compositional analysis of the eutectic high-entropy alloy were conducted using a Hitachi S-3400N scanning electron microscope (SEM) equipped with a tungsten filament, energy-dispersive spectroscopy (EDS), and an FEI Tecnai G2 F30 analytical transmission electron microscope (TEM) (Hitachi Company, Tokyo, Japan). The sample preparation process for XRD and SEM involved grinding the samples with SiC sandpaper, followed by fine polishing with a 1.5 μm diamond suspension. Subsequently, a mixed solution of 68% nitric acid and 37% hydrochloric acid in a volume ratio of 1:3 was used to etch the samples for 30 s. The TEM sample preparation process included cutting thin slices using electrical discharge wire cutting, mechanically grinding the slices to a thickness of 80–100 μm, then punching them into 3 mm diameter discs, further grinding the Φ3 mm discs to approximately 60 μm, and finally electrolytically thinning them to transparency using the same electrolyte and temperature (25 °C) as in electrochemical polishing, with a voltage of 25 V. Samples with dimensions of Φ 10 mm × 10 mm × 2 mm were obtained by wire cutting. The hardness of the alloy was measured using an HXD-1000 digital microhardness teste (Shanghai Optical Instrument Factory, Shanghai, China) with a testing load of 0.981 N and dwell time of 15 s. The compressive properties of the alloy at room temperature were tested using a Gleeble 3180 thermal simulation testing machine (Dynamic Systems Inc., New York, NY, USA) with sample dimensions of Φ 3 mm × 6 mm cylindrical specimens, a strain rate of 1 × 10^−3^ s^−1^, and a strain of 0.3. Nanoindentation creep tests were conducted using an Anton Paar Step300 nanoindenter (Anton Paar Company, Graz, Austria) to analyze the microdeformation mechanisms of the alloy. Samples with dimensions of Φ 10 mm × 10 mm × 2 mm were obtained via wire cutting, with equidistant points marked at 20 μm intervals. The peak load was kept constant at 10 mN for a holding time of 100 s, with three different loading rates of 0.1, 1, and 5 mN/s. Electrochemical corrosion tests were conducted on alloy samples with different compositions. The dimensions of the alloy samples used for electrochemical testing were 10 mm × 10 mm × 2 mm. Electrochemical specimens were prepared by soldering a copper conductor to the sample, embedding it in epoxy resin, and exposing a 10 mm × 10 mm testing area. The measurements were performed on a PGSTAT-302N electrochemical workstation using a standard three-electrode configuration, with a saturated calomel electrode (SCE) as the reference, a platinum electrode as the counter, and the alloy sample as the working electrode. Prior to testing, the specimens were immersed in the test solution for 30 min to stabilize the surface state. Electrochemical impedance spectroscopy (EIS) was carried out over a frequency range of 10^−2^ to 10^4^ Hz, with an AC perturbation amplitude of 10 mV. Cyclic polarization tests were initiated at −0.8 V and scanned to 1.5 V at a rate of 1 mV/s.

## 3. Results

### 3.1. The Effect of Zr Content on the Phase Structure and Microstructure of High-Entropy Alloys

**Figure 1:**
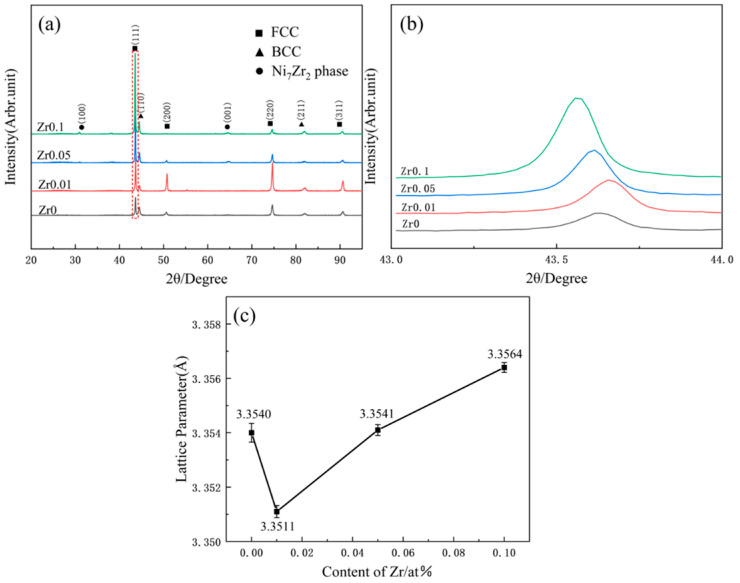
XRD pattern of the as-cast Zr_x_AlCoCrFeNi_2.1_ (x = 0, 0.01, 0.05, and 0.1) high-entropy alloys (**a**) XRD pattern; (**b**) (111) plane pattern; (**c**) line diagram of lattice parameters.

Figure 1 presents the XRD patterns of the as-cast Zr_x_AlCoCrFeNi_2.1_ (x = 0, 0.01, 0.05, 0.1) high-entropy alloys, along with the diffraction peaks of the (111) crystal plane. The results reveal that for x = 0, the alloy displays an FCC + B2 dual-phase structure (see Figure 1a). At x = 0.01, no new diffraction peaks appeared, while increasing the Zr content to x = 0.05 and x = 0.1 introduced new peaks at 2θ = 35.88° (100) and 2θ = 64.82° (001), which were previously reported by Wang et al. [15] and Li et al. [16], identifying Ni_7_Zr_2_. This peak, tentatively attributed to the Ni_7_Zr_2_ phase based on PDF card comparisons, required TEM verification to confirm its exact structure. Further analysis showed that the intensity of the diffraction peaks varied with the Zr content. Figure 1b shows that with the addition of Zr, the intensity of the (111) plane diffraction peaks gradually increased. Moreover, Figure 1c shows that the lattice parameters initially decreased, resulting in the diffraction peaks shifting to higher angles (to the right). As the Zr content increased further, the lattice constant also increased, causing the peak positions to shift leftward (toward higher angles). This shift is likely due to minor lattice changes in the solid-solution phase of the original AlCoCrFeNi_2.1_ high-entropy alloy with the addition of small amounts of Zr, similar to the influence of Nb on AlFeCrCoNi high-entropy alloys [17]. When Zr reached a certain level, new diffraction peaks appeared, indicating the gradual precipitation of Zr compounds from the B2 structure in the solid-solution phase. This precipitation increased the proportion of the precipitated phase while reducing the proportion of the B2 phase. Concurrently, Zr atoms were present in the FCC matrix as an interstitial solid solution, progressively strengthening the (111) crystal plane diffraction peaks. With further Zr addition, zirconium compounds precipitated more, leading to a noticeable shift in the diffraction peaks to larger angles.

**Figure 2:**
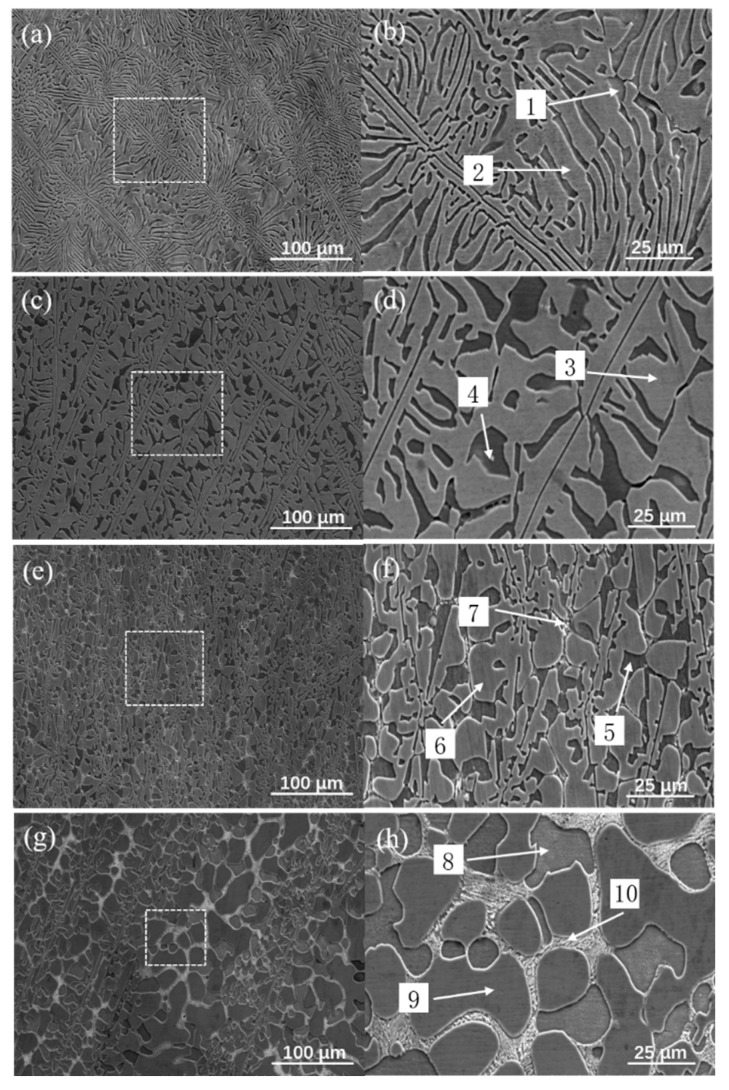
SEM images of as-cast Zr_x_AlCoCrFeNi_2.1_ alloys: (**a**,**b**) x = 0; (**c**,**d**) x = 0.01; (**e**,**f**) x = 0.05; (**g**,**h**) x = 0.1.

Figure 2 presents SEM images of the Zr_x_AlCoCrFeNi_2.1_ high-entropy alloy, illustrating the evolution of its microstructure with varying Zr content. In the absence of Zr (Figure 2a), the alloy predominantly consisted of FCC and B2 phases, exhibiting a distinct layered morphology. As the Zr content increased to x = 0.01 (Figure 2c), the microstructure began to change, indicating the coexistence of layered and petal-like structures. Here, the FCC phase transitions from a sheet-like to a petal-like form, accompanied by some degree of coarsening and dissolution; however, no precipitates are observed (Figure 2d). This behavior can be attributed to the very low Zr content, which dissolved into the FCC + B2 phase during solidification, resulting in lattice distortion and alteration of the phase structure of the alloy without significant precipitate formation. As the Zr content was further increased to x = 0.05 (Figure 2e), a notable transformation occurred in the alloy’s microstructure. The previously observed layered and petal-like structures completely evolved into petal-like structures [18]. Concurrently, zirconium compounds began to precipitate gradually between the FCC and B2 phases, primarily appearing as white particles that concentrated at the grain boundaries, thereby forming a discontinuous network structure (Figure 2f). This precipitation phenomenon is a result of the solidification process, where the primary FCC + B2 phase solidifies first, followed by secondary zirconium compounds from the residual liquid. Over time, the primary phase pushed the secondary phase to the grain boundaries, leading to the accumulation of zirconium compounds in these areas. When the Zr content increased further to x = 0.1 (Figure 2g,h), the petal-like morphology became more pronounced, and the accumulation of zirconium compounds at the phase boundaries intensified. This evolution is evident as the structural transitions from the discontinuous network observed in Figure 2e to a continuous network structure in Figure 2g.

To further clarify the phase composition of the alloys with varying Zr contents, micro-area composition analysis was performed using EDS. Table 2 shows the elemental composition of the high-entropy alloy based on EDS analysis. Without Zr, the composition in region 1 of the grain in Figure 2b closely matches the nominal composition. Based on the XRD results, region 1 corresponds to a B2-based phase, whereas region 2 represents an FCC-based phase. As the Zr content increased, the data in Table 1, along with regions 7 and 10 in Figure 2, indicated that the newly formed phase in Figure 2 was Ni- and Zr-rich, with an approximate 7:2 ratio. Therefore, it was inferred that the precipitated phase in Figure 2 is Ni_7_Zr_2_, which aligns with the XRD findings.

**Figure 3:**
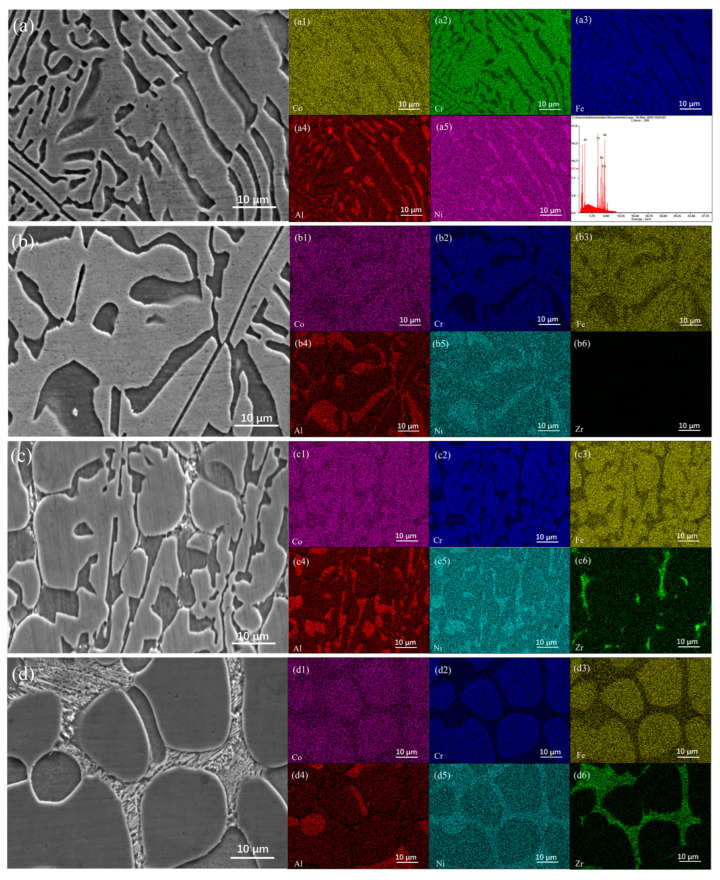
EDS mapping of as-cast Zr_x_AlCoCrFeNi_2.1_ alloys: (**a**) x = 0; (**b**) x = 0.01; (**c**) x = 0.05; (**d**) x = 0.1.

Figure 3 illustrates the elemental mapping distribution of the Zr_x_AlCoCrFeNi_2.1_ high-entropy alloy, providing insights into the compositional characteristics of the alloy. In the AlCoCrFeNi_2.1_ alloy, the distributions of Al, Co, Cr, Fe, and Ni were relatively uniform with minimal compositional segregation, as depicted in Figure 3a. However, upon addition of Zr, a distinct variation in the elemental distribution was observed. Figure 3b–d reveals that the FCC matrix phase becomes enriched in Al and Ni, whereas the B2 matrix phase shows a higher concentration of Co, Cr, and Fe. Additionally, the precipitated phases, which can be categorized as both granular and network-like, were rich in Ni and Zr. During the initial solidification, trace amounts of Zr preferentially dissolved into the FCC + B2 matrix. However, as the Zr content increased, the limited solubility of Zr led to its expulsion to the phase boundaries, resulting in regions that are enriched with Zr. With further increase in Zr content, a small fraction of Zr continued to dissolve into the FCC and B2 phases, while the remaining Zr contributed to the formation of the Zr-rich Ni_7_Zr_2_ phase within the AlCoCrFeNi_2.1_ matrix, as supported by previous studies [19]. When the Zr content reached x = 0.05, particles rich in Ni and Zr precipitated at the phase boundaries. These regions of enrichment began to form connections between the particles, suggesting the development of a discontinuous network structure, as illustrated in Figure 3(c1–c6). As the Zr content was further increased to x = 0.1, the precipitated phases coalesced to form a continuous network structure, highlighting the impact of Zr on the microstructural evolution of the alloy. To better understand the interactions between the alloying elements, Table 3 presents the atomic radii of the alloying elements and mixing enthalpies of the atomic pairs. Notably, Al and Ni exhibited significantly negative mixing enthalpies and shared the same crystal structure, which promoted mutual substitution at their respective lattice sites. This phenomenon indicates enhanced compatibility and may contribute to the observed enrichment of Al and Ni in the B2 phase [20]. Similarly, Co, Cr, and Fe possess comparable atomic radii and exhibit strong chemical compatibility, facilitating the formation of FCC phases enriched in these elements [21]. Moreover, the radius ratios of Zr to the elements in AlCoCrFeNi_2.1_ are as follows: R_Zr_/R_Co_ = 1.28, R_Zr_/R_Ni_ = 1.29, R_Zr_/R_Al_ = 1.12, R_Zr_/R_Cr_ = 1.25, and R_Zr_/R_Fe_ = 1.27, indicating minimal size disparity among the elements. In terms of electronegativity, Ni has a value of 1.91, which is significantly higher than that of the other elements, whereas Zr exhibits the lowest electronegativity in this alloy. This disparity suggests that a higher electronegativity corresponds to a greater tendency to attract electrons. Thus, among the alloying elements, Ni possesses a relatively strong electron affinity, whereas Zr has the least affinity. Furthermore, the similarity in the atomic radii of Zr and Ni favors the formation of the Ni_7_Zr_2_ phase, further elucidating the complex interactions within the alloy system.

**Figure 4:**
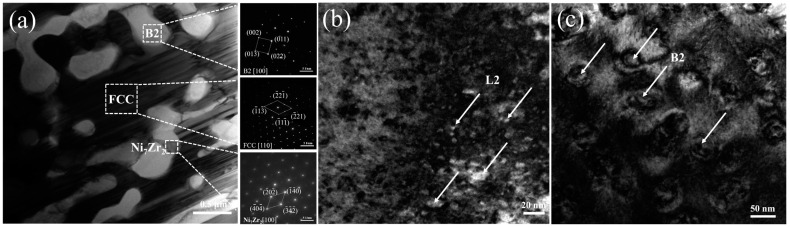
(**a**) TEM bright field image and corresponding SAED patterns of the three phases in cast Zr_0.1_AlCoCrFeNi_2.1_ alloy; (**b**) FCC phase TEM dark field image; (**c**) B2 phase TEM bright field image.

The high-resolution microstructure of the Zr_0.1_ alloy was analyzed using transmission electron microscopy (TEM). Figure 3 presents bright-field images of various regions in the Zr_0.1_ alloy, along with the associated selected-area electron diffraction patterns (SAEDs). In particular, the SAED pattern shown in Figure 4a reveals that the black region in the Zr_0.1_ alloy corresponds to the FCC phase, the white region is associated with the B2 phase, and the gray region represents the Ni_7_Zr_2_ phase, which is consistent with prior findings. In addition, the SAED pattern of the FCC phase shows faint diffraction spots in addition to the main FCC diffraction spots. These extra spots arose from the superlattice of the L1_2_ face-centered cubic ordered phase. Similar electron diffraction patterns for the FCC phase, which confirm that the black region is indeed the L1_2_ phase, were reported previously [23,24]. In this study, a supplementary super-diffraction spot from the FCC phase, indicated by a white circle, was chosen, and a dark-field image was acquired from this phase, as illustrated in Figure 4b. Spherical or ellipsoidal L1_2_ precipitates were dispersed within the FCC phase. Figure 4c shows a high-resolution bright-field image of the B2 phase, which reveals the presence of spherical nanoprecipitates. It is inferred that the addition of Zr encourages the formation of the Ni_7_Zr_2_ phase in the AlCoCrFeNi_2.1_ alloy and may also promote the precipitation of the L1_2_ ordered phase within the FCC phase. The nanoprecipitates in both the L1_2_ and B2 phases contribute to improved material performance by forming coherent or semi-coherent interfaces with the matrix phase. Nevertheless, excessive Zr segregation can result in embrittlement, particularly with the development of a continuous network of Zr-rich phases and zirconides. Therefore, optimizing alloy performance requires balancing the strengthening effects of these phases with the potential drawbacks of phase segregation. Further research on the mechanical properties will provide deeper insights into the performance variations with different Zr contents.

### 3.2. Influence of Zr Content on the Characteristics of High-Entropy Alloys

**Figure 5:**
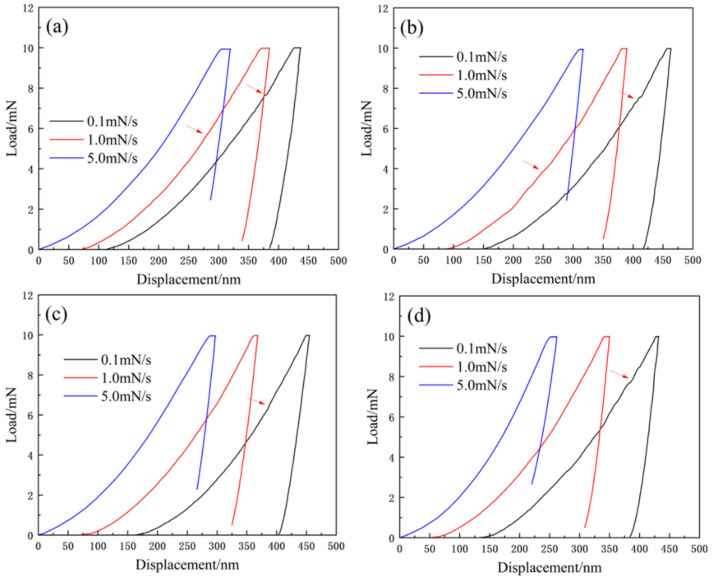
The load-displacement curves of the cast state Zr_x_AlCoCrFeNi_2.1_ alloy, with arrows indicating the pop-in phenomenon: (**a**) x = 0; (**b**) x = 0.01; (**c**) x = 0.05; (**d**) x = 0.1.

The FCC phase constitutes 70% of the original alloy and is the dominant crystalline phase [25], thereby exerting a more substantial influence on the 25 overall properties of the alloy. Analyzing the creep behavior of this primary phase can yield valuable insights into the comprehensive creep performance of the alloy. Nanoindentation techniques were used to examine the effects of grain boundaries, maximum load, and loading rate on the nano-creep behavior. As shown in Figure 5a–d, horizontal shifts were applied to the figures to differentiate between the curves. The experimental results revealed that, during the loading phase, the P-h curves at different loading rates were parallel, supporting the reliability of the data. In the holding stage, where the load remained constant, the displacement gradually increased, signifying creep behavior. After unloading, a minimal elastic recovery was observed, indicating that the alloy experienced permanent deformation. Comparing the maximum displacements of alloys with varying Zr content at an identical loading rate (e.g., 0.1 mN/s), the maximum displacements were measured as 270, 263, 241, and 247 nm, respectively. The maximum displacement of the alloys decreased with increasing Zr content. A similar trend was evident at loading rates of 1 and 5 mN/s, where the maximum displacement across all four alloys decreased as the loading rate increased. As indicated by the arrows in Figure 5a–d, a pop-in phenomenon appears in the P-h curves during the loading phase at a loading rate of 0.1 mN/s. This behavior reflects the response of the alloy to dislocation nucleation, with the nucleation sources typically being pre-existing dislocations or vacancies [26,27]. Notably, in comparison with the 1 mN/s loading rate, the pop-in points occur earlier and more frequently at a 0.1 mN/s loading rate. This is because, at lower loading rates, dislocations have ample time to nucleate and propagate, while the strain gradient is lower, facilitating dislocation slip [28]. As a result, the pop-in phenomenon occurs sooner and with a greater frequency.

Based on the load-displacement curves [29], the relationship between the creep displacement and time can be obtained through empirical fitting (with a confidence level greater than 95.6%):

(1) $$h=h_{0}+{a(t_{1}-t_{0})}^{b}+ct_{1}$$

where *h* represents the instantaneous indentation depth (nm), *h*_0_ is the indentation depth at the onset of creep (nm), *t*_1_ is the time (s), *t*_0_ is the time at the onset of creep (s), and *a*, *b*, and *c* are fitting parameters.

**Figure 6:**
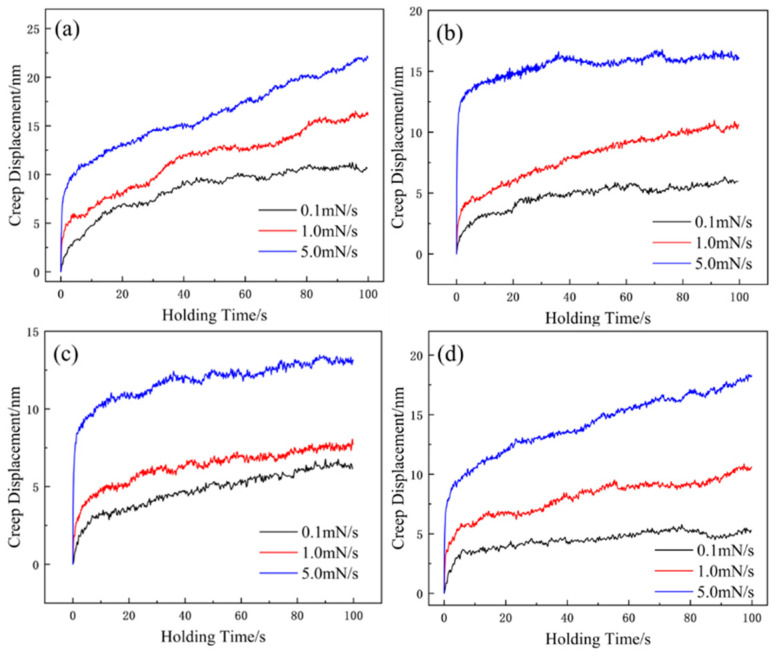
Creep displacement–time curves of as-cast Zr_x_AlCoCrFeNi_2.1_ HEAs: (**a**) x = 0; (**b**) x = 0.01; (**c**) x = 0.05; (**d**) = 0.1.

Figure 6a–d shows the creep displacement–time curves observed during the holding process. Unlike conventional creep curves, these curves display only two stages: transient and steady-state creep. At a loading rate of 5 mN/s, the creep depths of the alloy were 22.16, 20.39, 13.47, and 18.36 nm, respectively. These results indicate that as the Zr content increased, the creep resistance of the alloy improved to varying extents. This enhancement can be attributed to two main factors. First, the alloy possesses more slip systems within the FCC phase and a lower stacking fault energy. Zr addition stabilized the FCC phase, as shown by the increased intensity of the (111) diffraction peak in Figure 1b, which resulted in greater dislocation strengthening. Second, as the Zr content increases, the mixing enthalpy of the alloy becomes more negative (refer to Table 3), indicating a stronger entropy effect [30], which restricts atomic movement, thereby improving the creep resistance [31].

The equivalent stress and strain rate can be derived from the creep displacement–time curves [32]:

(2) $$\dot{\varepsilon }=\frac{1}{h}\frac{d_{h}}{d_{t_{1}}}$$

(3) $$\sigma \;=\frac{P_{\max}}{ch^{2}}$$

In this equation, the strain rate is represented as $\dot{\varepsilon }$, and the strain force is represented as σ, Pmax is the peak load, and *c* is the hardness indenter coefficient. For the Berkovich indenter, the value of *c* was 24.56 [33]. Similar to conventional alloys, the creep rate follows a power-law relationship with stress, and the creep stress exponent *n* is calculated using Equation (4) [34]:

(4) $$n\;=\frac{\partial \ln\dot{\varepsilon }}{\partial \ln\sigma }$$

**Figure 7:**
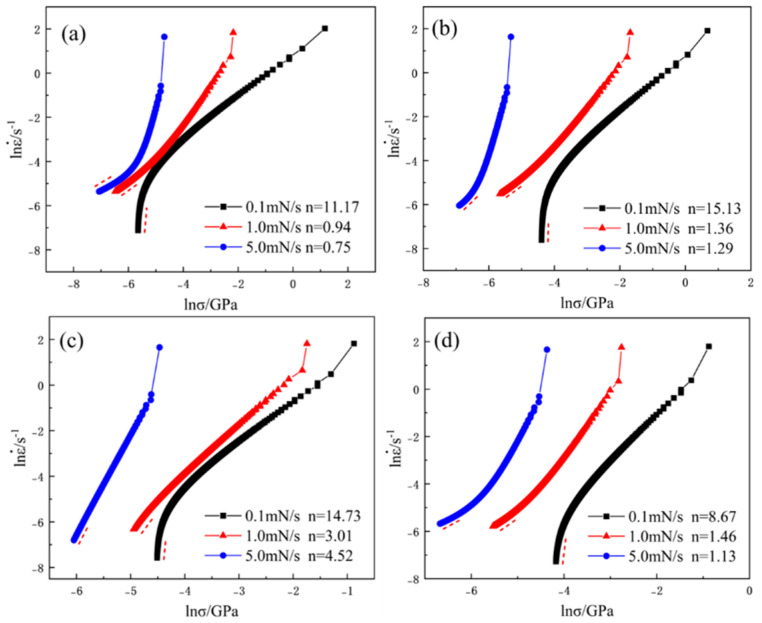
Creep stress exponent fitting curve of as−cast Zr_x_AlCoCrFeNi_2.1_ HEAs: (**a**) x = 0; (**b**) x = 0.01; (**c**) x = 0.05; (**d**) = 0.1.

Figure 7 shows the creep stress exponent curves of the alloy based on the fitted data. The creep stress exponent n is a key parameter for identifying the creep mechanism. When n < 1, the alloy undergoes diffusion creep; when 1 < n < 2, grain boundary creep occurs; and when n > 3, dislocation creep is the primary mechanism [35]. At a loading rate of 0.1 mN/s, the creep stress exponent of the Zr_x_AlCoCrFeNi_2.1_ alloy is greater than 3, indicating that creep deformation occurs via dislocation glide and climb. At a loading rate of 2 mN/s, the creep exponent significantly decreased. However, for the alloy with x = 0.05, the creep exponent n is 3.01, suggesting that this composition provides moderate strengthening, enabling grain boundary sliding, and resulting in good creep resistance with a high creep exponent. On the other hand, a higher Zr content (x = 0.1) causes excessive strengthening, grain boundary pinning, restricted dislocation motion, and embrittlement owing to phase transformation, leading to a decrease in the creep exponent. As the loading rate increases to 5.0 mN/s, the creep stress exponent n further decreases, as shown in Figure 7a,b,d. When the n value falls to approximately 1, the diffusion creep mechanism begins to dominate, indicating that a higher loading rate leads to stress concentration, with vacancy or vacancy-mediated nonhomogeneous dislocation nucleation acting as the initial mechanism for plastic deformation, followed by creep deformation through atomic-vacancy exchange [36]. As shown in Figure 7c, even at a loading rate of 5.0 mN/s, the creep stress exponent n remained greater than 3 when the Zr content was x = 0.05. This is due to the reduced presence of large-sized network phases, which favor the homogenization of the microstructure and effectively hinder grain boundary sliding and dislocation motion [37,38]. The addition of trace amounts of Zr further enhances the solid-solution strengthening of the alloy, thereby improving its creep resistance. Overall, different loading rates may trigger various deformation mechanisms within the alloy, and these mechanisms respond differently to varying Zr content, leading to changes in the creep exponent. This is due to the reduced presence of large-sized network phases, which favors the homogenization of the microstructure and effectively hinders grain boundary sliding and dislocation motion. The addition of trace amounts of Zr further enhances the solid-solution strengthening of the alloy, thereby improving its creep resistance. Overall, the effect of different Zr contents on creep performance provides a theoretical foundation for designing room-temperature creep-resistant materials, particularly for structural applications under low loading rates.

**Figure 8:**
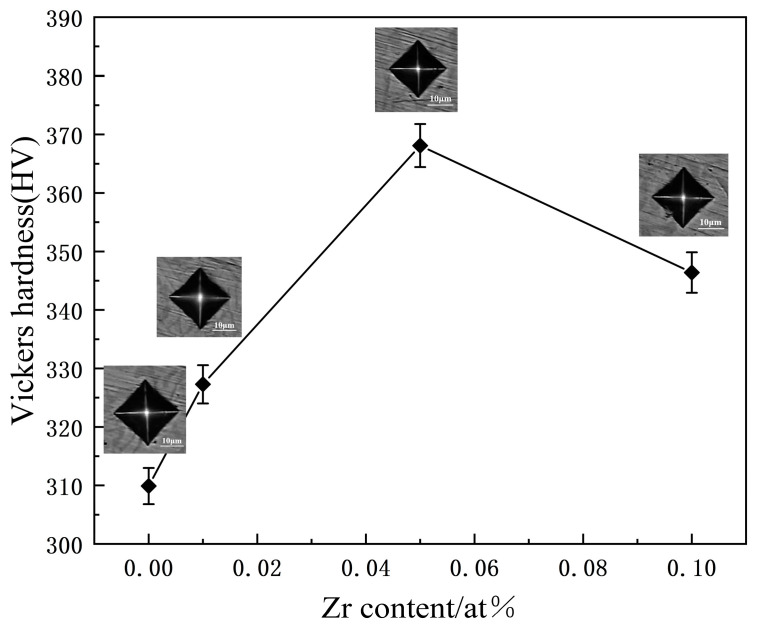
The relationship diagram of the hardness and lattice parameter of as-cast Zr_x_AlCoCrFeNi_2.1_ HEAs with the change of Zr content.

Figure 8 illustrates the average hardness and corresponding indentation morphology of the as-cast Zr_x_AlCoCrFeNi_2.1_ high-entropy alloys. The data revealed a notable trend: As the Zr content increased, the hardness initially increased before subsequently declining. Specifically, at a Zr content of x = 0.05, the hardness reached its peak value of 368.1 HV. This increase in hardness can be attributed to the effects of Zr, which leads to lattice distortion and enhanced solid-solution strengthening. In addition, the appropriate addition of Zr promotes the precipitation of fine Zr-based compounds (such as Ni_7_Zr_2_), which can effectively enhance the strength of the matrix [39]. Further analysis using XRD and EDS indicated that with the addition of Zr, some of the Zr reacted with Ni in the B2 phase to form the Ni_7_Zr_2_ phase. This phase contributed to the hardness of the alloy through precipitation strengthening, reinforcing the positive impact of Zr on the mechanical properties. However, as the Zr content further increased to x = 0.1, a significant change in the phase structure of the alloy occurred, as depicted in Figure 2b,h. The structure transitions from lamellar configuration to petal-like morphology. This transformation results in coarsening of the Ni_7_Zr_2_ phase, which has detrimental effects on the properties of the material. Coarsening not only weakens the grain boundaries but also depletes essential strengthening elements, such as Ni and Al, from the matrix, as shown in Figure 2d. Consequently, this reduction in the solid-solution and precipitation strengthening led to a decrease in the hardness [40]. In summary, while the initial addition of Zr enhances the hardness of the alloy through mechanisms such as solid-solution strengthening and precipitation hardening, especially when the Zr content is at x = 0.05, the hardness of the alloy reaches a maximum value of 368.1 HV. Excessive Zr content ultimately disrupts the microstructural integrity and reduces hardness owing to phase coarsening and depletion of critical alloying elements. This highlights the importance of optimizing the Zr content to achieve the desired mechanical properties in high-entropy alloys.

**Figure 9:**
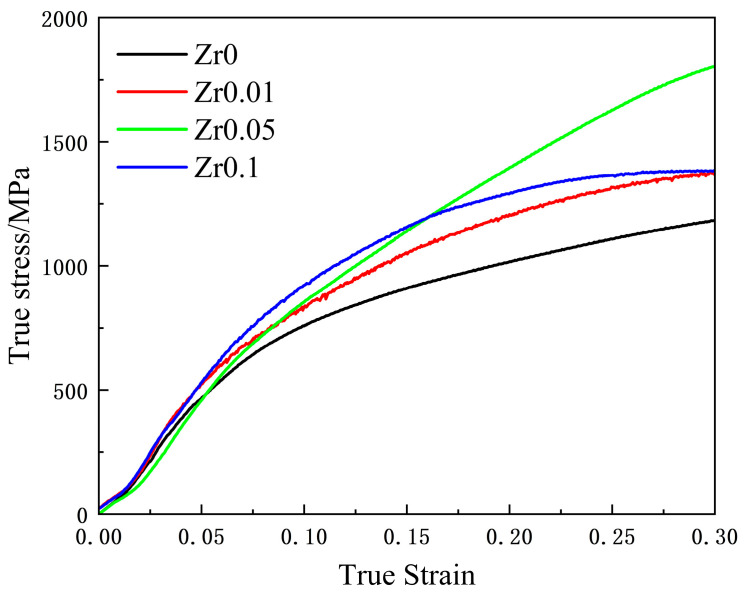
Room temperature stress–strain curves of as-cast Zr_x_AlCoCrFeNi_2.1_high entropy alloys.

Figure 9 presents the room-temperature compression stress–strain curves of the as-cast Zr_x_AlCoCrFeNi_2.1_ high-entropy alloys. The data revealed a clear trend: as the Zr content increased, both the compressive strength and yield strength of these alloys initially increased before subsequently declining. According to the specific data in Table 4, when the Zr content ranged from x = 0 to x = 0.05, the compressive strength of the alloy steadily increased. Notably, the compressive strength of the as-cast Zr_0_._05_AlCoCrFeNi_2.1_ high-entropy alloy improved significantly, reaching 618.4 MPa higher than that of the alloy without Zr. This enhancement in strength can be primarily attributed to the dominant plastic deformation mechanism during compression, which is dislocation slip [41]. The addition of Zr introduces substantial lattice distortion and leads to the formation of numerous particulate and discontinuous network precipitates dispersed within the grains (Figure 2f). As the mass fraction of the Ni_7_Zr_2_ phase increased, it maintained a coherent or semi-coherent relationship with the matrix. The elastic interaction between the coherent strain field and the strain field of moving dislocations results in coherent strain strengthening, ultimately providing the alloy with a higher strength [42]. However, when the Zr content was increased to x = 0.1, the compression strength of the alloy decreased to 1382.4 MPa, representing a reduction of 418.7 MPa compared to x = 0.05 alloy. This decline could be attributed to two factors. First, the formation of more continuous and coarse network zirconium compound precipitates in the alloy (see Figure 2h) alters the phase morphology and leads to coarsening of the phases, thereby weakening the precipitation strengthening effect [43]. Second, as the Zr content continues to increase, Figure 3(d1–d6) illustrates an increase in compositional segregation within the alloy, with Ni continuously precipitating in the B2 phase. This results in a reduction in the B2 phase and a corresponding decrease in the strength of the alloy. Furthermore, the yield strength data presented in Figure 4 indicate that the appropriate addition of Zr significantly enhances the yield strength of the alloy. In particular, when x = 0.05, the yield strength reached its peak at 1048.7 MPa, reflecting an increase of 48.7% compared to the alloy without Zr. In summary, the addition of Zr plays a crucial role in improving the compression strength of equiatomic AlCoCrFeNi_2.1_ alloy. This improvement is attributed to a notable synergistic effect between solid-solution strengthening from Zr atoms and precipitation strengthening from Zr compounds. The relationship between the Zr content and the mechanical properties underscores the importance of optimizing alloy composition to achieve enhanced performance. Overall, the ability to control the microstructure through Zr content opens up new avenues for tailoring high-entropy alloys with specific mechanical properties, enabling the development of high-strength and high-toughness materials.

**Figure 10:**
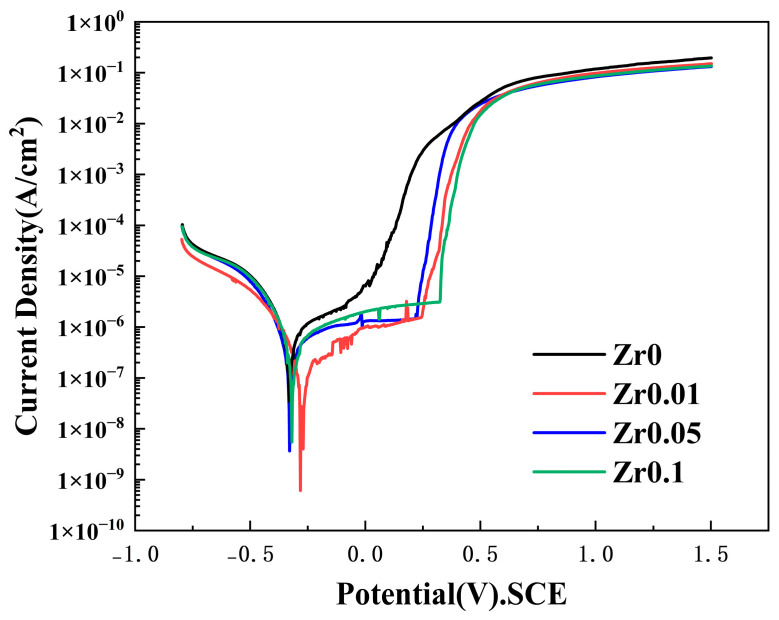
Potentiodynamic polarization curves of Zr_x_AlCoCrFeNi_2.1_ high entropy alloy in 3.5 wt% NaCl corrosion solution.

Figure 10 presents the linear sweep voltammetry (LSV) curve of the cast Zr_x_AlCoCrFeNi_2.1_ alloy in a 3.5 wt% sodium chloride solution. After soaking the sample in the solution for 30 min, a relatively stable potential was observed with fluctuations not exceeding 10 mV within 2 min. The LSV curve indicates that the alloy exhibits activation–passivation behavior in the solution. Moreover, no activation–passivation transition zone appeared in any of the polarization curves, suggesting the spontaneous formation of a stable passive film. The corrosion tendency of the cast Zr_x_AlCoCrFeNi_2.1_ alloy can be assessed based on the self-corrosion current density; a lower corrosion current density suggests less susceptibility to corrosion. The magnitude of the corrosion current reflects the corrosion rate of the eutectic high-entropy alloy; a higher corrosion current indicates a higher corrosion rate. Using the extrapolation method, the self-corrosion current density (Icorr) and self-corrosion potential (Ecorr) of the alloy were calculated, as shown in Table 5. The corrosion resistance of the alloys, in descending order, is x = 0.1 > x = 0.01 > x = 0.05 > x = 0.

This suggests that at x = 0.1, the alloy developed a more stable passive film, indicating that the corrosion resistance of the cast Zrx_0.1_AlCoCrFeNi_2.1_ high-entropy alloy improved with the inclusion of Zr. The introduction of Zr promoted grain refinement and increased the grain-boundary area. Meanwhile, the Ni_7_Zr_2_ phase precipitates, which tend to accumulate near the grain boundaries, increase with increasing Zr content. As the Zr content (x) rises from 0.01 to 0.1, the distribution of Ni_7_Zr_2_ changed from dispersed particles to a discontinuous network with a higher level of aggregation, eventually forming a continuous network structure. This leads to the formation of a more stable oxide film such as ZrO_2_ on the alloy surface. This oxide layer, exhibiting strong chemical inertness and barrier properties, effectively blocks corrosive ions like Cl^−^, thus enhancing surface corrosion resistance. Additionally, an increase in the number of precipitated phases reduced the Ni content in the matrix. Nickel, which is known for its corrosion-resistant properties, forms a NiO film that further prevents Cl^−^ penetration [44]. However, the alloy with x = 0.01 shows better corrosion resistance than that with x = 0.05, as the addition of zirconium at lower concentrations does not produce a fully uniform structure, resulting in localized precipitates and structural inhomogeneity. This may have primarily contributed to the increased susceptibility of the alloy to corrosion. At a Zr level of x = 0.05, the cast alloy exhibited the lowest self-corrosion potential and the highest self-corrosion current, indicating a preference for localized corrosion. To compare quantitatively the corrosion resistance across the alloys, the corrosion rates were calculated using specific formulas. Equations (5) and (6) show that when x = 0.1, the corrosion rate is minimized, reflecting the highest corrosion resistance. Compared to the Zr-free alloy, the corrosion rate decreased by an order of magnitude, demonstrating that optimal zirconium addition improves the corrosion resistance. As zirconium content increases, the corrosion resistance order is x = 0.1 > x = 0.01 > x = 0.05 > x = 0, aligning with the findings from corrosion current density measurements:

(5) $$K_{corr}=\frac{i_{corr}\cdot k\cdot EW}{\rho }$$

where *i_corr_* is the corrosion current density (A·cm^−2^), *ρ* is the mass density (g·cm^−3^), *EW* is the equivalent weight of the electrode (g), *k* is a constant with a value of 3272 mm/(A-cm-year), and *K_corr_* is the corrosion rate measured in millimeters per year (mmpy). The equivalent weight is the weighted average of the atomic weights of each element in the alloy, divided by the number of exchanged electrons (valence):

(6) $$EW=\sum \frac{f_{i}\cdot n_{i}}{A_{i}}$$

where $f_{i}$ is the mass fraction of the ith alloy component, $n_{i}$ is the number of exchanged electrons for the ith alloy component, and $A_{i}$ is the atomic weight of the *i*th alloy component (g/mol).

**Figure 11:**
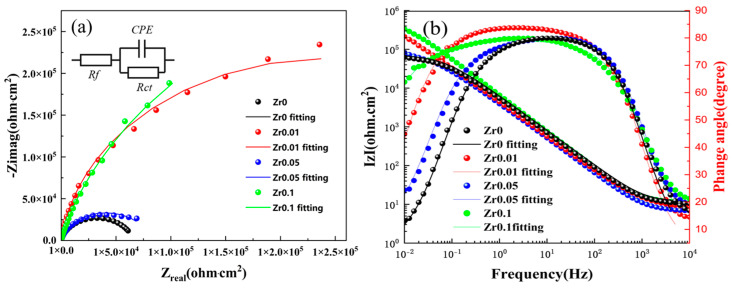
EIS images of the Zr_x_AlCoCrFeNi_2.1_ high entropy alloy in 3.5 wt% NaCl solution: (**a**) Nyquist plot and equivalent circuit diagram; (**b**) Bode plot.

Nyquist and Bode plots were utilized to analyze the impedance response, including magnitude and phase angle, for the cast Zr_X_AlCoCrFeNi_2.1_ high-entropy alloy in a 3.5 wt% NaCl solution. The simulated curves, generated using Nova 2.1 software, demonstrated strong agreement with the experimental data points, as shown in the Nyquist and impedance spectra (Figure 11a,b). The equivalent circuit model (Figure 11a) includes Rs, representing solution resistance, and Rct, corresponding to charge transfer resistance, with higher Rct values indicating greater resistance to electrochemical reactions. Additionally, the constant phase element (CPE) models the double-layer capacitance at the electrolyte–substrate interface, compensating for system heterogeneity. The mathematical expression for CPE impedance is as follows:

(7) $$Z_{CPE}=\frac{1}{{\left(j\omega \right)}^{n}Y_{0}}$$

where $Y_{0}$ is the proportional factor, and the value of n typically ranges from 0.5 to 1, reflecting the deviation of the double-layer capacitance from the ideal capacitance. When the value of n approaches 1, it indicates that the behavior of the constant phase element (CPE) is more similar to that of a capacitor, and the system is closer to an ideal capacitor.

As depicted in Figure 11, the Nyquist plot of the Zr_x_AlCoCrFeNi_2.1_ high-entropy alloy in a 3.5 wt% sodium chloride solution reveals that the Nyquist curves of the alloy tend to form semicircles, with their centers positioned below the x-axis. This could result from uneven surface diffusion or electrode polarization effects. A larger semicircle radius corresponds to a lower frequency and longer period, indicating a slower electrochemical reaction rate, which in turn implies better corrosion resistance of the alloy. Additionally, from the phase angle–frequency curve in Figure 11b, the high phase angle observed in the low-frequency region and the broad platform of the phase angle in the medium-frequency range indicate that the sample with x = 0.1 in Figure 11b, possesses a stable passivation film. In contrast, the sample with x = 0 exhibited a lower phase angle at low frequencies and a narrower phase angle platform, suggesting a reduced capacitance and a less stable passivation film. The values of n for each alloy in Table 6 show little variation and are all slightly less than 1, indicating that the double electric layer generated during the testing process is close to that of a pure capacitor. At the lowest frequency of 10^−2^ Hz, the |Z| value is approximately equal to the polarization resistance (Rct) value. As Rct increased, the difficulty of corrosion of the alloy also increased, indicating a stronger corrosion resistance. According to Table 5, the |Z| values of the cast Zr_x_AlCoCrFeNi_2.1_ alloys are arranged in descending order as follows: x = 0.1 > x = 0.01 > x = 0.05 > x = 0; this is consistent with the results discussed in the polarization curves above.

**Figure 12:**
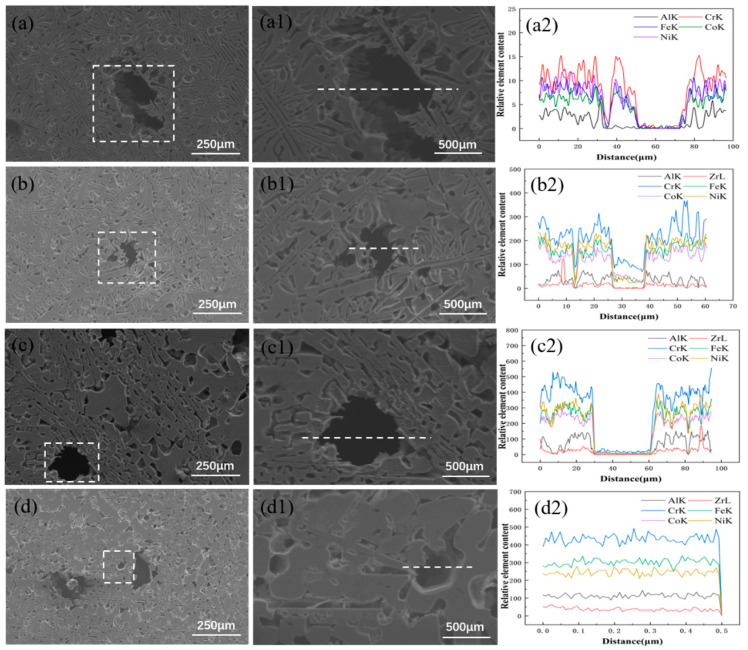
The surface pit morphology (in the boxes) and EDS line scan images (with the lines indicating the scan length) of the Zr_x_AlCoCrFeNi_2.1_ high entropy alloy after corrosion in 3.5 wt% NaCl solution: (**a**,**a1**,**a2**) x = 0; (**b**,**b1**,**b2**) x = 0.01; (**c**,**c1**,**c2**) x = 0.05; (**d**,**d1**,**d2**) x = 0.1.

As shown in Figure 12, both pitting and selective dissolution occurred in the Ni-Al rich B2 phase, whereas the Cr-rich FCC phase exhibited no significant corrosion phenomena. This can be attributed to the high aluminum content in the Ni-Al rich B2 phase, which results in an increased presence of Al_2_O_3_ within the passivation film on the B2 phase, thereby reducing the density of the film. Conversely, the passivation film on the Cr-rich FCC phase is comparatively denser, offering better protection [45]. It was demonstrated that in similar composition [46], the Al-Ni ordered BCC phase is more prone to forming micro-galvanic cells with other phases compared to the FCC phase. A higher proportion of the BCC phase increases the likelihood of preferential corrosion of the anode. When the Zr content (x = 0) is zero, the AlCoCrFeNi_2.1_ alloy without Zr exhibits significant corrosion pits and peeling areas in a corrosive environment, as shown in Figure 12a, The lack of effective corrosion-inhibiting phases and the uneven distribution of alloying elements in the matrix lead to the exacerbation of localized corrosion, especially with the enrichment of Ni and Cr in the corrosion pits [47]. These elements are more prone to forming corrosive galvanic cells, accelerating the occurrence of localized corrosion. At this point, owing to the lack of sufficient precipitates to hinder the corrosion reactions, the corrosion performance of the alloy is poor. When a small amount of Zr (x = 0.01) was added, the Ni_7_Zr_2_ precipitates began to form. Ni_7_Zr_2_ is a compound with high corrosion resistance, and it provides some localized protection in corrosive environments by reducing the active area of electrochemical reactions, thus decreasing the number of corrosion pits and improving corrosion performance. However, the distribution of Ni_7_Zr_2_ remains limited, and its protective effect on the entire alloy is insufficient to significantly improve the overall corrosion performance. As the Zr content increased to 0.05, the amount of Ni_7_Zr_2_ precipitate increased significantly. However, owing to its uneven distribution, regions enriched with Ni_7_Zr_2_ created an electrochemical potential difference with the surrounding matrix, leading to microgalvanic corrosion, especially at grain boundaries [48]. The accumulation of Ni_7_Zr_2_ exacerbated corrosion at the grain boundaries, resulting in more pronounced corrosion traces and pits, as shown in Figure 12c. From the corresponding EDS line scan curves, it can be observed that the content of each element in the alloy decreases simultaneously, without significant differentiation. This suggests that despite the increased amount of Ni_7_Zr_2_ precipitates, their uneven distribution lowers the overall corrosion performance of the alloy. When the Zr content increased to 0.1, the number and depth of the corrosion pits decreased significantly, and the surface corrosion morphology became more uniform and dense, as shown in Figure 12d. The Ni_7_Zr_2_ precipitates are more uniformly distributed and elements such as Ni and Cr contribute to the formation of a stable oxide film. While Zr may form ZrO_2_ within the oxide layer, a dense oxide film forms at the interface between Ni_7_Zr_2_ and the matrix, which can effectively block the intrusion of corrosive media. Additionally, when x = 0.1, a large number of continuous network structures of the corrosion-resistant Ni_7_Zr_2_ precipitate phase aggregate within the alloy, thereby reducing the area fraction of the matrix phase and decreasing the extent of the easily corrodible regions. This indicates that an appropriate amount of Zr not only enhances the alloy’s corrosion resistance through precipitation strengthening but also improves surface protection by promoting the uniform distribution of precipitates and reducing the easily corrodible phases. Overall, the enhanced corrosion resistance at x = 0.1 provides valuable insights for the development of corrosion-resistant materials, particularly for applications in high-salt or acidic environments such as marine engineering and chemical equipment.

## 4. Conclusions

(1) In the Zr-containing alloy, the FCC phase is enriched with Co, Cr, and Fe, the B2 phase is enriched with Al and Ni, and the Laves phase is enriched with Zr and Ni. Additionally, the FCC phase includes L1_2_ precipitates, while the B2 phase contains BCC precipitates. As Zr content increases, the alloy’s microstructure transitions gradually from a lamellar to a petal-like structure. With higher Zr levels, Ni_7_Zr_2_ precipitates progressively emerge within the B2 phase, resulting in a continuous reduction of the B2 phase and an increase in the Ni_7_Zr_2_ phase. At x = 0.01, the Ni_7_Zr_2_ phase has a dispersed granular form; at x = 0.05, it takes on a discontinuous network pattern; and at x = 0.1, it develops into a continuous mesh structure.

(2) The suitable addition of Zr can greatly enhance the alloy’s creep resistance. At a loading rate of 0.1 mN/s, the alloy’s creep stress exponent n exceeds 3, suggesting that under low stress and slower loading rates, the primary creep mechanism in the alloy is dislocation creep. At x = 0.05, the alloy exhibits dislocation creep at different creep rates, showing robust creep resistance. At this content, Zr promotes microstructural homogenization, hinders grain boundary sliding, enhances solid-solution strengthening, and improves creep resistance. When the Zr content is x = 0.1, it causes excessive strengthening, grain boundary pinning, restricted dislocation motion, and embrittlement due to phase transformation, leading to a decrease in the creep exponent.

(3) With the increase in Zr content, the compressive strength, yield strength, and hardness of the alloy show an overall upward trend, reaching a maximum at x = 0.05. At this point, the yield strength increases by approximately 48.7% to 1048.7 MPa, the compressive strength increases by approximately 52.2% to 1801.1 MPa, and the hardness increases by approximately 18.7% to 368.1 HV. The synergistic effect of solid-solution strengthening by Zr atoms and precipitation strengthening by hard zirconium compounds is highlighted.

(4) In the high-entropy alloy AlCoCrFeNi_2.1_ (x = 0, 0.01, 0.05, 0.1), when x = 0.1, the alloy exhibits the lowest self-corrosion current density of 0.034197 μA/cm^2^, with a corrosion potential of 277.55 mV, indicating optimal corrosion resistance. This is primarily due to the amount and uniformity of the Ni_7_Zr_2_ precipitates in the alloy, which are key factors affecting the corrosion resistance of the Zr_x_AlCoCrFeNi_2.1_ high-entropy alloy. At a Zr content of x = 0.1, Ni_7_Zr_2_ can achieve a uniform distribution, forming a dense protective layer and oxide film, thereby enhancing the overall corrosion resistance of the alloy.

## Figures and Tables

**Table 1:** Nominal and actual composition of as-cast Zr_x_AlCoCrFeNi_2.1_ alloy (at%).

Content	Element						Total
	Al	Co	Cr	Fe	Ni	Zr	
X = 0	8.51	18.59	16.40	17.61	38.88		
	8.63	18.63	16.99	17.97	37.78		
X = 0.01	8.49	18.54	16.35	17.57	38.77	0.287	100
	8.57	18.12	16.87	17.60	38.44	0.39	100
X = 0.05	8.39	18.33	16.17	17.37	38.33	1.42	100
	8.19	18.23	16.53	17.30	38.43	1.32	100
X = 0.1	8.27	18.07	15.94	17.12	37.79	2.80	100
	7.83	17.87	16.26	16.84	37.82	3.38	100

**Table 2:** The chemical composition distribution of cast Zr_x_AlCoCrFeNi_2.1_ high-entropy alloy (at%).

	No.	Elements					
		Al	Co	Cr	Fe	Ni	Zr
X = 0	1 (BCC)	28.60	13.00	9.53	11.32	37.55	
	2 (FCC)	11.68	17.18	20.02	17.85	33.27	
X = 0.01	3 (BCC)	27.59	12.53	9.24	10.70	39.62	0.30
	4 (FCC)	10.20	18.19	20.29	19.02	32.10	0.19
X = 0.05	5 (BCC)	25.24	13.38	10.02	11.42	39.54	0.73
	6 (FCC)	10.64	18.03	19.92	18.94	32.29	0.17
	7 (Ni_7_Zr_2_)	15.13	15.38	14.02	15.67	31.81	7.99
X = 0.1	8 (BCC)	25.59	13.46	10.42	11.48	38.72	0.32
	9 (FCC)	10.63	18.16	20.49	19.91	30.66	0.15
	10 (Ni_7_Zr_2_)	14.53	14.23	10.53	10.94	39.55	10.53

**Table 3:** Electronegativity, atomic radius, and binary mixing content of various elements [22] (kJ·mol^−1^).

	Al	Co	Cr	Fe	Ni	Zr
Electronegativity of elements	1.61	1.88	1.66	1.83	1.91	1.33
Atomic radius(pm)	143	125	128	126	124	160
Al		−19	−10	−11	−22	−44
Co			−4	−1	0	−41
Cr				−1	−7	−12
Fe					−2	−25
Ni						−49

**Table 4:** Parameters of friction and wear samples at room temperature.

Zr Content	x = 0	x = 0.01	x = 0.05	x = 0.1
Yield strength (MPa)	705.1 ± 31.53	740.8 ± 12.56	1048.7 ± 23.84	865.9 ± 14.85
Compressive strength (MPa)	1182.7 ± 24.68	1372.3 ± 10.26	1801.1 ± 28.70	1382.4 ± 13.56

**Table 5:** Electrochemical corrosion parameters of Zr_x_AlCoCrFeNi_2.1_ high-entropy alloy in 3.5 wt% NaCl solution.

Zr Content	Ecorr (mV)	Icorr (μA·cm^−2^)	Epit (mV)	Kcorr (mm/year)
x = 0	−332.06	0.09993	20.27	0.0011621
x = 0.01	−318.01	0.097945	244.83	0.0011381
x = 0.05	−330.08	0.098589	224.97	0.0011456
x = 0.1	−277.55	0.034197	323.06	0.00039737

**Table 6:** The equivalent circuit parameters of the EIS for the Zr_x_AlCoCrFeNi_2.1_ high-entropy alloy.

Zr Content	Rs (Ω·cm^2^)	Rct (Ω·cm^2^)	CPE	
			Y0 (F/cm^2^)	n
x = 0	10.1	62,600	3.35 × 10^−5^	0.899
x = 0.01	11.0	486,000	2.78 × 10^−5^	0.930
x = 0.05	6.53	78,000	4.90 × 10^−5^	0.891
x = 0.1	8.85	700,000	4.59 × 10^−5^	0.872

## Data Availability

The original contributions presented in this study are included in the article. Further inquiries can be directed to the corresponding author.
